# Green Tea: Current Knowledge and Issues

**DOI:** 10.3390/foods14050745

**Published:** 2025-02-22

**Authors:** Maya Radeva-Ilieva, Stanila Stoeva, Nadezhda Hvarchanova, Kaloyan D. Georgiev

**Affiliations:** Department of Pharmacology, Toxicology and Pharmacotherapy, Faculty of Pharmacy, Medical University—Varna, 9002 Varna, Bulgaria; stoeva.st@mu-varna.bg (S.S.); nadejda.hvarchanova@mu-varna.bg (N.H.); kaloyan.georgiev@mu-varna.bg (K.D.G.)

**Keywords:** green tea, catechins, epigallocatechin-3-gallate, pharmacokinetic, bioavailability, interaction

## Abstract

Green tea possesses antioxidant, anti-inflammatory, anticancer, and antimicrobial activities, reduces body weight, and slows down aging. These effects are primarily attributed to catechins contained in green tea leaves, particularly epigallocatechin-3-gallate. However, in humans, the realization of green tea’s beneficial effects is limited. In order to summarize and critically analyze the available scientific information about green tea’s health benefits and issues related to its use, we conducted an in-depth literature review in scientific databases. A number of in vitro studies reported that green tea catechins modulate various signaling pathways in cells, which is thought to underlie their beneficial effects. However, data on the effects of catechins in humans are scarce, which is partly due to their low stability and oral bioavailability. Furthermore, catechins may also participate in pharmacokinetic interactions when co-administered with certain drugs such as anticancer agents, drugs for cardiovascular diseases, immunosuppressors, etc. As a result, adverse drug reactions or therapy failure may occur. In conclusion, over the years, various approaches have been investigated to optimize catechin intake and to achieve beneficial effects in humans, but to date, the use of catechins for prophylaxis or disease treatment remains limited. Therefore, future studies regarding the possibilities of catechins administration are needed.

## 1. Introduction

Green tea, derived from the leaves of the *Camellia sinensis* (L.) Kuntze plant, is among the most widely consumed and health-promoting beverages worldwide. Its use dates back to around 3000 BC in ancient China. Historical records, including the ancient medical text *Shen Nong’s Herbal Classic*, demonstrate that the Chinese population was aware of tea’s health-promoting and disease-preventative properties. Subsequently, the recognition of tea’s health benefits has transcended national boundaries, becoming widely acknowledged both within China and internationally [1]. Currently, tea cultivation has expanded to over 30 countries, but is enjoyed by over 3 billion people across 160 countries and regions. The primary nations recognized for their significant tea production include China, India, Sri Lanka, Japan, Taiwan, and Kenya [1,2]. As a result, tea contributes significantly to global economic growth. In 2020, the global tea market was valued at approximately USD 200 billion, and it is projected to increase to over USD 318 billion by 2025 [3].

In recent years, green tea consumption has become very popular due to its flavor and appealing fragrance, and for its numerous health-beneficial effects as well as enhancing overall well-being. A number of in vitro and in vivo studies have reported its antioxidant, anti-inflammatory and antimicrobial effects, anticancer activity as well as cancer prevention, anti-aging effect and weight loss [4,5].

Various biologically active substances (BAS) are present in tea leaves: polyphenolic compounds, alkaloids, polysaccharides, saponins, free fatty acids, vitamins, minerals and others [6,7]. It is assumed that green tea’s health benefits are due to the polyphenolic compounds, and in particular the catechins, contained in green tea leaves. A lot of catechins are detected in tea leaves but the following catechins are usually present in greater amounts: (−)-epigallocatechin-3-gallate (EGCG), (−)-epicatechin-3-gallate (ECG), (−)-epigallocatechin (EGC) and (−)-epicatechin (EC). Among these, EGCG is the predominant catechin in tea leaves. Moreover, EGCG is believed to be the most active catechin compound responsible for most of green tea’s beneficial effects [8,9,10,11,12,13]. It is generally thought that the impact of catechins primarily stem from the modulation of critical signaling pathways and transcription factors (nuclear factor kappa B (NF-kB) signaling pathway, activator protein 1 (AP-1) transcription factor, mitogen-activated protein kinase (MAPK) signaling pathway, nuclear factor erythroid 2-related factor 2 (NRF2), signal transducer and activator of transcription 1 (STAT1) transcription factor, and others), which in turn affect gene and protein expression in cells [4,14,15,16,17]. Currently, the therapeutic use of catechins is limited due to their low oral bioavailability and poor stability. However, various strategies are being explored to address these challenges. A key approach involves incorporating catechins into liposomes, nanoparticles, or other drug delivery systems, which may improve their stability, gastrointestinal absorption, and plasma concentrations. Studies have shown that these methods have the potential to enhance the in vivo effectiveness of catechins, including EGCG [18,19,20].

It should also be noted that green tea composition may vary significantly due to several factors, including the growing conditions of the tea plants, the timing of the leaf harvest, and post-harvest processing as well as storage conditions. These variations in BAS content are also linked to different organoleptic characteristics of both the tea leaves and the final tea beverage after preparation [21,22]. Furthermore, the type of green tea—such as Chinese, Japanese, Korean, and others—can differ based on the geographical conditions in which the tea plants are cultivated, and this also influences the qualitative and quantitative levels of BAS [23,24].

The purpose of this article was to present current trends and potential problems associated with green tea use. Therefore, we comprehensively analyzed the latest scientific information about green tea properties and health benefits as well as methods of green tea consumption. To gain a better understanding of the possible benefits of green tea’s health-beneficial effects, we conducted a thorough literature search in the PubMed, Web of Science and Scopus databases and collected a number of articles, mostly published in the last ten years. The search was performed using the following keywords: green tea, Japanese green tea, Chinese green tea, processing, manufacturing, steaming, catechins, epigallocatechin-3-gallate, interactions, pharmacokinetics, bioavailability, health benefits, antioxidant, anti-inflammatory, anticancer.

## 2. Green Tea Varieties

Tea is obtained from the dried leaves of the plant *Camellia sinensis* (L.) *Kuntze*, *Theaceae* [25]. *C. sinensis* is divided into two main varieties: *assamica*, known for its larger tea plant and leaves (up to 20 cm in length), and *sinensis*, which has smaller leaves (5–12 cm) [26]. Although they are harvested from the same plant, there are many different types of tea depending on the manufacturing process. Fresh tea leaves undergo various processing techniques. The most common classification of these tea types is based on their origin, complex production processes and similarity of final products, leading to six widely recognized tea types: white, green, oolong, yellow, black and dark teas [5]. 

Depending on the degree of endogenous enzymatic reaction (by the polyphenol oxidase and peroxidase contained in the leaves), the six types of tea are grouped into five categories: (1) non-fermented tea: green tea—produced by drying and steaming fresh leaves to deactivate polyphenol oxidase, no oxidation occurs; (2) lightly fermented teas: yellow tea and white tea; (3) semi-fermented tea: oolong tea—produced by partially fermenting fresh leaves before the drying process; (4) fully fermented tea: black tea—that undergoes fermentation after harvesting, before the drying and steaming process; and (5) post-fermented tea: dark tea, where external microbial fermentation is crucial to the processing [8,27]. 

Among all of these main tea varieties, however, the most significant effects on human health have been observed with the consumption of green tea [28]. It is one of the most consumed tea varieties worldwide and has been steadily gaining popularity, now accounting for about 20% of global tea production. The majority of green tea production is concentrated in China and Japan [5,29]. However, other countries such as Korea, India, Sri Lanka, Kenya, Vietnam, and Indonesia are also associated with green tea production but not to such an extent [30,31].

China’s extensive history of green tea cultivation, combined with its vast production regions, diverse tea cultivars, and varied processing techniques, has resulted in thousands of green tea products. The principal green tea varieties in China include Longjing, Maofeng, Huangshanmaofeng, Biluochun, Xinyangmaojian, Gunpowder, Chun Mee, Lung Ching, Mao Feng, among others [32,33]. 

Similarly, Japan offers a wide array of green tea varieties, with an estimated 20 distinct types recognized for their diversity. Notable varieties include Sencha, Bancha, Matcha, Gyokuro, Hojicha, Kamairicha, Kukicha, Kariganech, Konch and Genmaicha [32]. Each variety is characterized by specific processing techniques and distinct flavor profiles. Sencha and Bancha/Genmaicha are the most commonly consumed types of green tea in Japan. Sencha is made from leaves collected during the first and second harvests (first or second flushes of green tea), while Bancha is produced from tea leaves harvested in late summer or early autumn, a method known as late seasonal picking (third or fourth flush of green tea). Genmaicha is a blend of Bancha and roasted brown rice [34,35]. 

## 3. Green Tea Production and Composition

### 3.1. Green Tea Production

**Figure 1:**
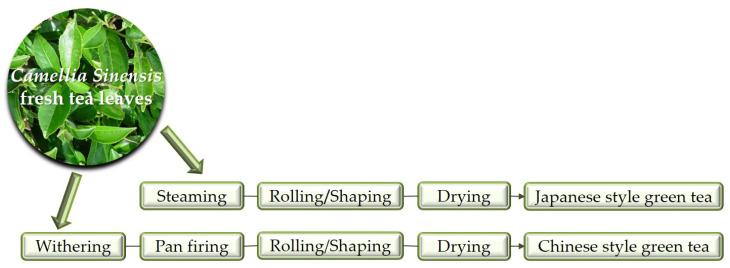
Major steps for green tea processing.

Green tea is prepared from whole leaves. Its production begins with an initial heating step that inactivates oxidizing enzymes. This step effectively preserves the catechins, maintaining the leaves’ green color and delicate flavor. The next crucial step is rolling, where the leaves are cut and twisted to release plant juices and coat the leaf surface. Subsequently, the leaves are dried to reduce moisture content, resulting in a dried tea product [29]. Two distinct processing methods are employed for the heating step (Figure 1). In Japanese-style green tea (e.g., Sencha) fresh tea leaves are steamed at high temperatures (100 °C) in large rotating cylinders for 20–50 s [36,37]. In contrast, Chinese-style green tea involves rapid roasting or pan-firing (heating in a pan over a flame) at higher temperatures (around 300–350 °C) [30]. However, pan-firing can also be used for certain Japanese-style green teas like Kamairicha, a premium variant [38]. There are notable differences in the rolling stage between Japanese and Chinese green teas. Chinese style green teas are typically rolled unidirectionally, while Japanese styles are rolled bidirectionally. Additionally, some Chinese style green teas undergo a withering process, which is omitted in manufacturing. This omission might be due to the limited capacity of tea processing facilities when large volumes of fresh tea leaves need to be processed [39]. The final form of green tea depends on the specific variant being produced. Green tea is primarily made from the leaves of *Camellia sinensis var. sinensis*. In contrast, the Assam variety (*Camellia sinensis var. assamica*) contains high levels of polyphenols, resulting in an excessively bitter taste [29]. Furthermore, the diverse methods of green tea production are bound to result in compositional differences. Therefore, it is essential that reports on feeding studies or in vitro experiments explicitly outline the tea processing techniques utilized. However, the composition of green tea closely resembles that of the fresh leaf, particularly in terms of its primary components.

### 3.2. Green Tea Composition

The composition of green tea is well documented and represents a complex mixture of many compounds. Phytochemical studies indicate that green tea contains approximately 4000 naturally occurring bioactive chemical compounds, which are secondary metabolites [40]. These precious compounds include polyphenols (predominantly catechins), carbohydrates, alkaloids, organic acids, pigments, amino acids and numerous flavor compounds. A wide variety of other components also exists, including saponins, lignins, flavones, depsides, lipids (linoleic and linolenic acids), minerals, vitamins and enzymes [41,42,43]. The production of polyphenolic compounds, such as catechins, by *Camellia sinensis* is directly influenced by the environmental and agricultural conditions in which it is cultivated. Notably, the antioxidant activity of green tea infusions increases with rising temperature. The composition of green tea varies depending on a range of environmental factors, including cultivation conditions, soil, climate, and external influences such as light, geographical location, microorganisms, and temperature [44]. Horticultural practices, the age of tea trees and leaves, as well as the technological processes involved in tea production, are all crucial factors. For optimal tea cultivation, a tropical or subtropical climate is essential, with a temperature range of 13 °C to 29 °C and an altitude of 2460 m above sea level. The soil should maintain a pH between 4.0 and 5.5, be well-drained, and contain over 2% organic matter [45]. Additionally, the soil should ideally be sandy loam or sandy clay enriched with humus. For crops that require high nitrogen levels, studies have shown that the application of ammonium-based fertilizers can significantly enhance growth performance and yield. This practice not only boosts the levels of free amino acids and caffeine but also modulates the polyphenol content, potentially either increasing or decreasing it [46]. The application of nitrogen, potassium and magnesium fertilizers has been shown to enhance the synthesis of enzymes such as glutamic-pyruvic transaminase (GTP), glutamate dehydrogenase, and amine oxidase, leading to an increase in amino acids. Consequently, this practice results in a reduced polyphenol/amino acid (PP/AA) ratio, an index historically used to assess tea quality [46,47]. Previous studies have indicated that the application of nitrogen and phosphorus fertilizers can increase the concentrations of theanine, caffeine, and esterified catechins, thereby enhancing the quality of tea infusions [48,49]. Furthermore, various micronutrients play essential roles in the physiological processes of tea plants by functioning as electron transports, enzyme activators or cofactors. For instance, iron is crucial for the synthesis of chlorophyll-protein complexes, while polyphenol oxidase in tea leaves contains copper [50]. Additionally, tea trees are characterized by their ability to hyperaccumulate aluminum and manganese. Research has indicated that aluminum may be beneficial for tea root growth, showing high tolerance in environments with high aluminum availability and low pH conditions [51]. Hence, the antioxidant properties, which significantly affect polyphenolic levels, are modified as a defensive mechanism against oxidative stress [52,53].

Green tea is typically prepared by steeping the tea leaves in hot water (80–90 °C) for 3–4 min [54]. Water temperature and infusion time significantly influence the extraction efficiency of BAS such as polyphenols and methylxanthines, with the majority of EGCG and caffeine being extracted at a water temperature of 80 °C. Moreover, differences in extraction rates and BAS content can be observed when comparing the infusion of bulk tea leaves to that of powdered substances [30,54]. Regarding extraction time, some studies have shown that the total amount of extracted polyphenols increases with longer infusion times (≥10 min). However, this also leads to increased astringency and bitterness, affecting the organoleptic properties of the tea beverage [30,55]. For example, in a study on Japanese Bancha green tea, it was found that extending the steeping time from 5 to 30 min extracted a greater amount of polyphenols but also increased tannin extraction, contributing to the unpleasant astringent taste of the brewed tea [56]. Additionally, as infusion time and temperature rise, catechins may undergo chemical changes such as epimerization, where epicatechins convert to their corresponding catechins. EGCG has been found to epimerize into GCG at temperatures above 85 °C. Unlike catechins, caffeine is a stable molecule that readily dissolves in hot water, maintaining relatively stable concentrations in the tea beverage. Furthermore, according to Saklar et al. (2015), the largest amounts of EGCG and caffeine were extracted at a water temperature of around 85 °C in an infusion lasting 3 min [54].

#### 3.2.1. Polyphenols

The primary components of green tea are polyphenols, which constitute 25–35% of the dry weight of green tea leaves. Polyphenols are one of the most defining features of the tea plant and have been studied more extensively than any other class of compounds in tea; they contribute to its taste, particularly the bitterness and astringency [57]. Polyphenols represent about 30 varieties of compounds, mainly composed of flavonoids, anthocyanins and phenolic acids [58]. 

##### Catechins

**Figure 2:**
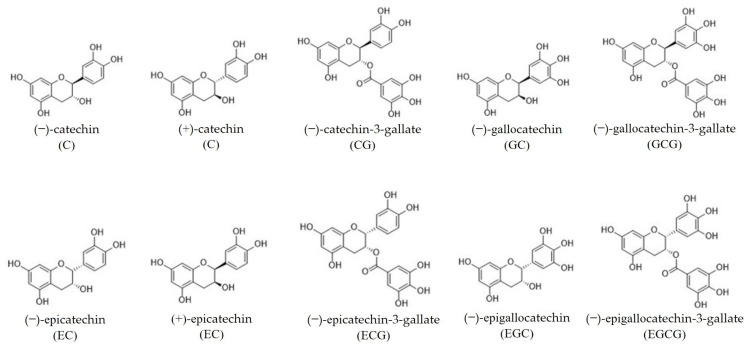
Chemical structures of major green tea catechins.

Catechins are the main polyphenolic constituent in green tea, comprising 70% of the total polyphenolic content and may reach 30 to 42% of the dry weight of tea leaves [9,11]. Catechins belong to a more general class of flavonoids known as flavan-3-ols, which are also referred to as flavanols. They are the most significant of all tea components [33]. Green tea plants contain various catechins, with EGCG being the most abundant, making up about 50–70% of the total [43,59]. This is followed by EGC at approximately 19%, ECG at around 13.6%, and EC at roughly 6.4%. Catechin and gallocatechin are also found in lesser amounts [8,14,41,42]. The chemical structures of major catechins are presented in Figure 2. It is observed that juvenile leaves contain higher levels of EGCG and ECG compared to mature leaves, while older leaves exhibit increased levels of EGC and EC [60]. The abovementioned concentrations of different catechins can be influenced by multiple factors. The preparation techniques impact the catechin content both quantitatively and qualitatively. Additionally, the catechin levels in the original tea leaves vary based on factors such as variety, origin, and growing conditions [61]. Fresh green tea preparation does not completely extract catechins from the leaves, therefore, the concentrations observed differ from those determined through complete extraction methods [62,63].

##### Anthocyanins

Anthocyanins, a class of water-soluble pigments, are part of the flavonoid family. Although their concentration in tea is relatively low, their bitter taste significantly impacts the quality of tea. Examples of anthocyanins include cyanidin, delphinidin, pelargonidin, malvidin, and petunidin [33].

##### Flavonols

Flavonols, another class of polyphenols present in tea leaves, predominantly exist in the form of glycosides, rather than as their aglycones (non-glycosylated) form [64,65]. Green tea is rich in flavonol glycosides, which constitute 2–3% of the tea infusion. The primary ones are myricetin, quercetin, and behenyl glycosides, with their sugar chains composed of monosaccharides like glucose, galactose, rhamnose, and arabinose, as well as disaccharides and trisaccharides. Due to their lower polarity, aglycones tend to remain within the plant matrix during water extraction. Consequently, they are not present in significant quantities in tea beverages [66]. 

##### Phenolic Acids

Although the phenolic acid content in green tea is relatively low, it encompasses a variety of compounds such as chlorogenic acid, gallic acid, caffeic acid, p-coumaric acid, quinic acid, ellagic acid and tea gallate [67]. 

#### 3.2.2. Carbohydrates

Tea contains minor amounts of monosaccharides and disaccharides, including glucose, fructose, galactose, and sucrose. The majority of the carbohydrates (5–7% dry weight) in tea are polysaccharides. Next to polyphenols, tea polysaccharides (TPS) are another principal bioactive component of tea. It has been observed that the polysaccharide content in tea increases as the raw tea leaves mature, which is notably different from the pattern observed in tea polyphenols [68]. Furthermore, TPS exhibit a wide range of chemical characteristics. These include their monomer composition (e.g., glucose, galactose, rhamnose, arabinose, xylose and mannose), their acidity (which can be neutral or acidic), their solubility (whether they are water-soluble or not), and their conjugation with proteins, polyphenols, metal ions, and selenium. These factors significantly influence the structure-–function relationship [69,70,71,72]. Due to the diversity in tea species, processing technologies, and the methods employed for isolating and purifying TPS, over 120 TPS with varying chemical characteristics have been documented. Studies suggests that TPS may have potential benefits, including immune-regulatory, antioxidant, anticancer, anti-obesity and antidiabetic effects [33,69]. The diverse bioactivities of TPS are intrinsically linked to their structural characteristics, which encompass their chemical composition, the configuration and location of glycosidic bonds, molecular weight, molecular weight distribution, and chain conformation. These structural features are unequivocally and significantly influenced by the types of tea materials used, the processing technologies employed, the extraction methods applied, and any modifications made during these processes [73]. For instance, TPS with lower polyphenol content exhibit greater antioxidant activity compared to those with higher polyphenol content. Furthermore, integrating selenium can markedly enhance the antioxidant properties of TPS [74].

#### 3.2.3. Alkaloids

Fresh tea leaves typically contain 3–4% of alkaloids, recognized as methylxanthine, including caffeine (theine), theobromine, and theophylline. Among these, caffeine is the most abundant, comprising 2–5%, while theophylline and theobromine are present in smaller quantities [75,76]. Historically, tea has been cherished for its caffeine content. Caffeine is considered a significant component of tea, providing mood and cognitive-enhancing benefits [44,77]. 

#### 3.2.4. Amino Acids

Tea comprises approximately 1% to 4% amino acids, which are associated with its quality [78]. To date, 26 amino acids have been identified in tea, including 20 protein amino acids and six non-protein amino acids. The highest concentrations are found of theanine, aspartic acid, arginine, alanine, glutamic acid and tyrosine [8]. Theanine, a nonproteinic amino acid found exclusively in tea, constitutes about half of the total amino acid content and is associated with giving tea its unique umami flavor [27,75].

#### 3.2.5. Volatile and Non-Volatile Aroma Compounds

The aroma of green tea primarily comes from volatile aromatic compounds, which constitute about 0.005% to 0.020% of the chemical components. Their variety is quite complex. Numerous studies have analyzed the volatile components of green tea, leading to the discovery and identification of more than 600 compounds. Some examples of aromatic compounds include dimethyl sulfide, pentanal, hexanal, hexadecane, heptanal, acetone, acetic acid, etc. [79]. Additionally, more than 40 organic acids are present in green tea, significantly contributing to its aroma and taste. Some of these acids are volatile compounds, including acetic acid, hexenoic acid and butyric acid. Others are non-volatile organic acids, such as oxalic acid, tartaric acid, formic acid, L-malic acid, citric acid, and ascorbic acid, among others [32]. 

#### 3.2.6. Saponins

To date, the saponins reported from the genus *Camellia* are primarily acylated pentacyclic triterpenoid saponins, and most of them belong to the oleanane-type triterpenoid saponins [80]. Tea saponins are primarily extracted from *C. Oleifera* seed, although the leaves of *C. sinensis* are also valuable sources, containing 12 specific saponins. Among them are Floratheasaponin A, Theasaponin B1, Foliatheasaponin, Isotheasaponin (B1, B2, B3). These saponins mainly consist of sapogenins, glycosides, and organic acids. They have been associated with a broad spectrum of health benefits [80,81].

#### 3.2.7. Minerals, Vitamins and Others

The minerals found in greatest quantities in green tea are phosphorus and potassium, followed by calcium, magnesium, iron, manganese, aluminum, sulfur, and silicium and trace elements such as zinc, copper, and fluorine [82,83]. Additionally, green tea contains various vitamins, including vitamin C, E, K, B2 and B3, also enzymes (e.g., glucosidases and lipoxidases), and a variety of pigments, including chlorophyll B, β-carotene, pheophorbide A and xanthophyll [66,84]. 

## 4. Pharmacokinetic Green Tea–Drug Interactions

Considering the rich composition of biologically active substances described above, green tea has long-proven health benefits; however, in some cases, these compounds could result in undesirable interactions with concomitantly used drugs. Some published reviews conclude that there is generally no clinically significant risk of such interactions, but nevertheless recommend caution, especially for drugs with a small therapeutic index [85,86]. The focus will primarily be on interactions at the pharmacokinetic level, as they are more challenging to understand and predict, and the precise mechanisms have not been adequately explored or confirmed. The best studied and reported of the pharmacokinetic ones are those related to the absorption, decreased bioavailability and modification in biotransformation of the victim drug, so the main focus will fall on them [87,88].

### 4.1. Pharmacokinetic Interactions of Green Tea at the Level of Absorption

Due to the low bioavailability of green tea catechins, it is assumed that the involved interactions are primarily in the gastrointestinal tract, particularly in enterocytes, in a manner similar to how grapefruit juice interacts. The transporters and metabolizing enzymes present in this area may be influenced or regulated, and direct physicochemical interaction is not excluded either. 

#### 4.1.1. Interactions Related to SLC (Solute Carrier) Transporters

The organic anion-transporting polypeptides (OATPs) family, primarily OATP1A2 (SLCO1A2), OATP2B1 (SLCO2B1), OATP1B1 (SLCO1B1), and OATP1B3 (SLCO1B3), is involved in the disposition of numerous drugs and other xenobiotics. OATP1A2 and OATP2B1 are ubiquitously expressed, e.g., at the blood–brain barrier, testis, renal proximal tubule cells, prostate, etc., but also at the apical membrane of enterocytes, and play an important role in absorption, whereas OATP1B1 and OATP1B3 are found primary at the basolateral membrane of hepatocytes [89]. Important substrates which may fall victim to interactions with them include steroid hormones, bile acids, statins, antihypertensives, antibiotics, antifungals, and chemotherapeutic agents [90,91]. The catechins that are present in green tea, such as ECG and EGCG, appear as substrates, but show modulatory activity with respect to these transporters as well. Inhibitory effects are reported with OATP1A2, OATP1B1, and OATP2B1, while OATP1B3-mediated uptake is stimulated [92]. Clinical studies conducted with healthy volunteers that co-administered green tea extract or EGCG with drug substrates of OATP1A2 (e.g., rosuvastatin, atorvastatin, nadolol, lisinopril) displayed reduced levels of maximum plasma concentration (C_max_) and area under the curve (AUC) [93,94,95,96]. 

#### 4.1.2. Interactions Related to ABC (ATP-Binding Cassette) Transporters

The other location where absorbed drug levels can be affected is modulation of efflux pumps. P-glycoprotein (P-gp; MDR 1, ABCB 1) was the first of these pumps to be cloned and therefore is the best studied of them. P-gp is expressed in numerous barrier and excretory epithelial tissues, including intestinal enterocytes, hepatocytes, renal tubule cells, and brain capillaries, where it is localized primarily in apical membranes, but also on tumor cells, where it is associated with the development of resistance [97,98,99]. It plays a crucial role in the systemic distribution of and exposure to lipophilic and amphipathic drugs, carcinogens, toxins, and other foreign substances [100]. Green tea extracts and particularly EGCG have contradictory effects on P-gp function. Most data from in vitro and in vivo studies point to inhibitory effects on P-gp [85,101,102,103]. This inhibition of P-gp, which reduces the risk of cancer cells developing resistance, and many other effects of green tea constituents are seen as positive in oncology. However, possible pharmacokinetic interactions should be taken into account to avoid changes in antitumor drugs and to exploit the benefits of green tea use. A similar case exists with concomitant administration of erlotinib, lapatinib or sunitinib with green tea extract or EGCG, where pharmacokinetic parameters of tyrosine kinase inhibitors (such as AUC_0–∞_ and C_max_) were significantly reduced [104,105]. The authors rule out the possibility of a direct physicochemical interaction between green tea and tyrosine kinase inhibitors, but do not provide precise explanations for the possible mechanisms that cause the reduced bioavailability. Veerman et al. (2022) explain the interaction between nintedatib, a multi-target tyrosin kinase inhibitor used as first-line monotherapy for idiopathic pulmonary fibrosis (IPF), and green tea with P-gp induction as the most probable mechanism [106]. A similar increase in P-gp activity by the catechins in green tea has been described in an earlier work by Wang et al. (2002), which concluded that it was due to activation of allosteric sites [107].

#### 4.1.3. Other Interactions

Direct physicochemical interaction and/or decreased solubility are proposed mechanisms in interactions of green tea with amoxicillin, raloxifene and bortezomib [108,109,110,111]. Catechins, as polyphenol compounds, can lead to change in the extent of dissolution due to formation of insoluble complexes when co-administrated with amine, piperazine or piperidine group-containing drugs [110,111,112,113]. In the case of bortezomib, a drug used in the treatment of multiple myeloma, the interaction has been demonstrated in vitro on myeloma cells and resulted in a decrease in bortezomib’s activity after conformational changes occurred, yet it can be assumed that concomitant use in the gastrointestinal tract would further complicate the interaction.

### 4.2. Pharmacokinetic Interactions of Green Tea at the Level of Biotransformation

#### 4.2.1. Interactions Related to Cytochrome P450 Enzymes (CYP450)

Not less confusing is the effect of green tea on drug-metabolizing enzymes, mainly cytochrome P450 enzymes (CYP450). Data from in vitro studies or from studies in experimental animals indicate that green tea possess inhibitory effects on the major drug-metabolizing cytochrome enzymes, such as CYP 1A2, 2C9, 2D6 and 3A4. However, some studies reported that green tea may induce CYP450 activity [114,115,116]. Nevertheless, studies conducted in healthy volunteers show no significant changes in plasma concentrations of selective substrates of these enzymes [117,118,119]. The cited studies in healthy volunteers were conducted either with decaffeinated tea infusions [118] or with individual catechins, e.g., EGCG [119]. The latter study hinted at a 20% inhibition of CYP3A4 by EGCG, but still concluded that there was no clinical significance. Based on this, we developed our hypothesis that the interactions with CYP450 enzymes associated with the use of green tea could be driven not only by the catechin fraction, but also due to the mutual potentiation with other biologically active components in the infusion, such as the methylxanthine fraction [76]. Methylxanthines, represented mainly by caffeine, and less by theophylline and theobromine, are significantly less-studied than catechins; they could also confer an increased risk of interactions, not only as perpetrator agents but also as victims. They are eliminated mainly by CYP1A2 metabolism and can be influenced by inducers and inhibitors as victims, but they can also disrupt the metabolism of other substrates of this form and be perpetrators [120]. In addition, other cytochrome isoforms, such as CYP2D6 and CYP3A4, could also be affected by methylxanthines [76,121].

#### 4.2.2. Interactions Related to UDP-Glucuronosyltransferase (UGT)

Other enzymes that are also involved in biotransformation of green tea catechins are uridine 5′-diphospho-glucuronosyltransferases (UGTs). UGTs activity could also be modified by green tea catechins. The data here also point to both inhibitory [122,123] and inductive effects [124] on these enzyme systems. The latter study discusses the possibility of interaction of green tea with the antitumor agent irinotecan and its active metabolite (SN38), whose inactivation is linked to UGT1A1 activity. However, the conclusion was that there is no considerable risk of interaction between green tea and the inactivation of irinotecan.

### 4.3. Databases of Potential Green Tea–Drug Interactions 

An increasing number of databases of drug–drug interactions are beginning to add dietary supplements and herbal remedies, indicating that their intake is not devoid of the risk of such interactions and should be taken into account by the attending physicians. One of the most commonly used drug–drug interaction databases is Lexicomp^®^ Drug interaction [125]. It has reported a total of 48 interactions related to the use of green tea and, according to this database, green tea is classified in the group of inducers of P-gp, together with carbamazepine, rifampicin, St John’s Wort and other strong inducers. Therefore, most specified interactions are with drug substrates of P-gp, such as dabigatran etexilate, sirolimus, digoxin, aliskiren, tenofovir alafenamide, etc., and would be affected by concurrent use with an inducer. For most of them no reference source of direct mutual influence is given, and it is assumed that since it is a strong inducer of P-gp, it would behave like the others of this group. With some of the aforementioned drugs, such as the tyrosine kinase inhibitors erlotinib, lapatinib or sunitinib, no interactions were detected in the database, while the interaction with nintedanib was classified based on the aforementioned work in risk rating group B (no action needed) [106].

From the review, it can be concluded that there is a risk of pharmacokinetic interactions between green tea and certain drugs, but at this time it remains unclear if it is clinically relevant. Assessing pharmacokinetic drug–herb interactions with natural products is challenging due to the complexity of these products, which contain numerous components whose composition can vary based on factors such as the quality of the herbal drug, preparation method, dosage, dosage form, and the timing and duration of administration. As a result, the observed effects may be diverse and unclear, necessitating individual case monitoring.

## 5. Green Tea Health Benefits and Possible Mechanism of Action

**Figure 3:**
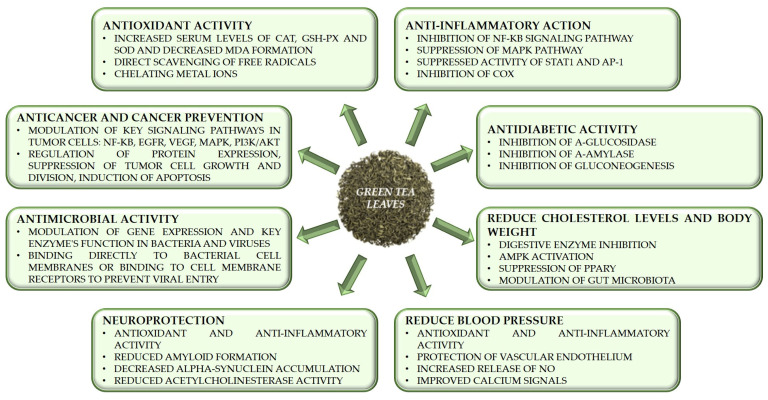
Beneficial effects of green tea leaves and putative mechanisms of action [2,15,16,17].

Several beneficial effects are attributed mainly to the catechins contained in green tea leaves (Figure 3).

### 5.1. Antioxidant Effects

One of the most extensively studied effects of tea polyphenols, particularly catechins, is their antioxidant activity, which has been observed both in vitro and in vivo, including in clinical trials. Individual tea polyphenols exhibit different antioxidant properties [4,14]. It is known that their biological activities, including antioxidant potential, are influenced by their structure, particularly the number and position of hydroxyl groups within the molecule. In this regard, EGCG has been identified as the most potent compound, believed to be responsible for most of the biological effects attributed to catechins [13,126,127,128].

Several key mechanisms are thought to contribute to the antioxidant potential of catechins. These include increasing the levels of endogenous antioxidant enzymes by regulating their expression, inhibiting lipid peroxidation, directly scavenging free radicals, and chelating metal ions to suppress oxidative reactions [129]. Research has shown that catechins’ antioxidant activity is linked to an increase in serum levels of antioxidant enzymes such as catalase (CAT), glutathione peroxidase (GSH-Px), and superoxide dismutase (SOD), alongside a reduction in malondialdehyde (MDA) formation, which is a byproduct of lipid peroxidation and a marker of oxidative stress [129]. Antioxidant enzymes play a key physiological role by inactivating free radicals, which are normal byproducts of cellular metabolism, including reactive oxygen species (ROS) and reactive nitrogen species (RNS). Under normal conditions, there is a balance between free radical generation and their removal within the cell. When this balance is disrupted, an increase in free radical production occurs. These unstable and highly reactive compounds can damage lipids, proteins, and nucleic acids, leading to oxidative or nitrosative stress and cellular damage. Furthermore, catechins can directly neutralize free radicals by interacting with their reactive forms, resulting in the formation of relatively stable phenolic oxygen radicals. Catechins also have the ability to chelate metal ions (Fe^2+^, Cu^2+^, Ca^2+^), which can catalyze oxidation reactions, thereby forming inactive complexes and preventing the initiation of lipid peroxidation [4,14,130].

### 5.2. Anti-Inflammatory Effect

Catechins exhibit anti-inflammatory activity associated with modulation of key signaling pathways and transcription factors in the cell such as the NF-kB and MAPK signaling pathways, AP-1 transcription factor, STAT1 transcription factor and others. This is associated with influencing gene expression and protein expression in cells [4,15,16,17,131,132]. It is suggested that inhibition of the NF-kB signaling pathway is of fundamental importance for the anti-inflammatory activity of catechins [132,133]. The NF-kB signaling pathway is involved in a number of cellular processes, including inflammation, immune response, cell proliferation, differentiation and death. This signaling pathway is tightly regulated and its dysregulation is associated with the development of certain diseases. Activation of the NF-kB signaling pathway is observed in inflammatory reactions, tumor diseases and disorders of the immune system [127,134,135]. Moreover, catechins also inhibit other signaling pathways in the cell such as the MAPK signaling pathway, resulting in suppressed activation of extracellular signal regulated kinase (ERK), c-Jun N-terminal kinase (JNK) and p38 proteins as well as inhibition of AP-1 activity. It is also reported that catechins suppress the activation of STAT1 transcription factor which is also involved in the initiation of the inflammatory response. The end result of cell cascade modulation is reduced production of proinflammatory cytokines such as tumor necrosis factor alpha (TNF alpha), interleukins (IL-1, IL-6, IL-8) and others [3,16,17]. Additionally, there is evidence that EGCG inhibits the activity of cyclo-oxygenases (COX), reducing their expression due to the influence on various cellular signaling pathways, which probably contributes to its anti-inflammatory activity [127,131]. Furthermore, catechins can induce the activation of the Keap1/Nrf2/ARE signaling pathway, which is important for antioxidant defense mechanisms but may also be related to the anti-inflammatory action [17]. Recently, Kuang et al. (2022) hypothesized that catechin plays an anti-inflammatory effect through mediating ferroptosis [136]. However, despite the different molecular mechanisms of catechins’ anti-inflammatory action observed in vitro, their action in vivo is not fully understood yet.

### 5.3. Anticancer Activity

Numerous studies have demonstrated that catechins are beneficial in preventing and treating cancer. EGCG, in particular, has been identified as a potential agent for preventing cancer development [137,138]. Many preclinical studies have focused on assessing the antitumor properties of green tea extract, catechins, and EGCG, as well as exploring the mechanisms behind these effects. Clinical research has also examined the antitumor effects of EGCG [59]. Although the exact mechanism remains unclear, it is believed that catechins exert their antitumor effects through their antioxidant properties, induction of apoptosis, regulation of the cell cycle, modulation of cell signaling pathways (such as the NF-kB pathway), protein expression, and enzyme activity, including that of drug-metabolizing enzymes. These mechanisms likely contribute to cancer prevention and the stimulation of physiological functions [4,127,137]. Additionally, many preclinical studies have shown that EGCG interacts with several key cell signaling pathways in tumor cells, including NF-kB, EGFR, VEGF, MAPK, and PI3k/AKT. As a result, EGCG regulates protein expression, suppresses tumor cell growth and division, induces apoptosis, and promotes cell death by blocking cell cycle progression [59,127,139]. EGCG has also been found to inhibit cyclo-oxygenase 2 (COX-2) enzyme activity, primarily through suppression of the MAPK pathway, which is linked to its effects on the cell cycle and cancer cell death [127,139]. Moreover, CYP450 is involved in phase I metabolic reactions, converting procarcinogens into harmful intermediates that can bind to DNA, lipids, or proteins and initiate free radical formation. In contrast, phase II enzymes detoxify the cell through conjugation with endogenous substrates. Studies suggest that catechins inhibit CYP450 enzymes, preventing procarcinogen activation while boosting the activity of phase II detoxifying enzymes. These processes are thought to contribute to their antitumor, antioxidant, and anti-inflammatory effects [4,139]. Additionally, tea polyphenols have been reported to inhibit tyrosine kinases, which could enhance the effectiveness of tyrosine kinase inhibitors used in cancer therapy [104]. EGCG may also serve as an adjuvant to chemotherapy. Synergistic antiproliferative and pro-apoptotic effects were observed when EGCG was combined with tamoxifen in human breast cancer cell lines, both in vitro and in vivo. Similar synergistic effects have been noted when EGCG was paired with other antitumor agents like doxorubicin and paclitaxel [59,127,138].

### 5.4. Antimicrobial Activity

Green tea and its catechins possess both antibacterial and antiviral properties [18,128]. Numerous in vitro studies have demonstrated the antibacterial effects of catechins against various bacteria, including both gram-positive (such as *Staphylococcus aureus*) and gram-negative strains (like *Escherichia coli*, *Pseudomonas aeruginosa*, *Helicobacter pylori*), including multidrug-resistant *staphylococci*. Additionally, catechins have shown synergistic effects when combined with antibacterial medications. These compounds affect bacterial cells both directly and indirectly, potentially through altering gene expression, inhibiting key enzymes, and binding directly to bacterial cell membranes. This binding may increase membrane permeability, generate hydrogen peroxide (H_2_O_2_), and cause damage to the membrane, preventing the bacteria from attaching to host cells. Catechins have also been found to inhibit bacterial toxin activity [18,140,141,142]. Furthermore, EGCG has been shown in vitro to reduce plaque formation and inhibit the growth of *Streptococcus mutans*, a major contributor to dental caries, likely through modulation of gene expression and protein production [141]. 

Catechins also exhibit antiviral properties against various DNA and RNA viruses in laboratory settings. Their antiviral action may stem from EGCG binding to cellular proteins, disrupting their function, interacting with cell membrane receptors to prevent viral entry, or affecting gene expression [128,143]. Sinecatechins, a fraction derived from green tea extract, have been approved by the U.S. FDA for the topical treatment of external genital and perianal warts caused by the human papillomavirus. This fraction contains 85-95% catechins, with over half being EGCG, and is used for immunocompetent adults over 18 years old [9,144,145].

In addition to these effects, recent studies have shown that EGCG can inhibit the replication of SARS-CoV-2, the virus responsible for COVID-19, which has caused a global pandemic. The progression of COVID-19 varies widely among individuals, ranging from asymptomatic cases to severe complications like respiratory failure, organ damage, and life-threatening coagulation disorders. While vaccines have been developed to prevent infection or lessen its severity, there are few antiviral drugs specifically targeting SARS-CoV-2, and some are still in development. As research continues, established drugs and plant-based compounds like EGCG are being explored for their potential in treating COVID-19. EGCG’s antiviral effect may be linked to its interaction with the ACE2 receptor, necessary for viral entry, or its inhibition of viral proteases and RNA-dependent RNA polymerase, both of which are crucial for viral replication. However, while EGCG shows promise, it is not yet considered a primary therapeutic agent for COVID-19 due to its lack of specificity but could be considered a complementary treatment, particularly in the early stages of the infection to stimulate the physiological functions [146,147,148].

### 5.5. Reducing Cholesterol Levels and Managing Body Weight

Numerous studies have shown that green tea extract promotes weight loss and improves glucose and lipid metabolism, particularly in obese individuals with metabolic syndrome [149]. Regular green tea consumption is linked to a reduced risk of developing type 2 diabetes and cardiovascular complications associated with diabetes [150]. A meta-analysis by Yuan et al. (2018) of randomized controlled trials demonstrated that green tea consumption lowers plasma levels of total cholesterol and low-density lipoprotein (LDL) in overweight and obese individuals [151].

Various mechanisms have been proposed to explain the effects of green tea on body weight. Some researchers suggest that weight loss may be due to changes in gut microbiota or the inhibition of digestion and absorption of macronutrients (lipids, proteins, carbohydrates), possibly through the suppression of digestive enzymes and transmembrane transporters. Another suggested mechanism is that catechins, including EGCG, activate adenosine monophosphate-activated protein kinase (AMPK), which regulates the expression of proteins involved in cellular metabolism, thus explaining many of the metabolic effects of green tea [152,153].

Additionally, catechins have been found to reduce lipid accumulation in preadipocytes, inhibit their differentiation, and induce apoptosis in mature adipocytes, potentially through AMPK activation or modulation of other signaling pathways [149,154]. Catechins also inhibit the activity of the COMT enzyme, leading to increased levels of endogenous noradrenaline, which promotes fatty acid oxidation and thermogenesis [154].

### 5.6. Other Effects

Research has demonstrated that both green tea and EGCG possess neuroprotective properties. Regular green tea consumption has been linked to a reduced risk of developing Alzheimer’s disease, Parkinson’s disease, and dementia, particularly among Asian populations. Additionally, green tea and EGCG inhibit amyloid formation during aging, which may help alleviate cognitive decline associated with amyloid accumulation [155,156]. Furthermore, green tea consumption is associated with a reduced risk of cardiovascular disease, as it lowers LDL levels, reduces blood pressure, and improves endothelial function [153].

## 6. Factors Influencing the Bioavailability of Green Tea Catechins (GTCs) and Strategies for Addressing Associated Challenges

The role of catechins in relation to flavor quality and the health benefits of green tea is globally acknowledged. In this regard, the increased interest in the natural treatment of many chronic and degenerative diseases has drawn attention to the compounds in question and prompted reasonable efforts to standardize their intake [157,158]. It should be mentioned that the concentration of catechins in infusions obtained from dried plant material was found to be approximately 3.83 ± 0.6 g/L [23]. Notably, only a remarkably small fraction of these compounds (approximately 1.68%) can be absorbed and, consequently, be bioavailable following oral consumption [159,160]. In humans, the maximum plasma concentrations achieved are a mere 1–2 μmol/L, occurring between one and two hours post-consumption, followed by a rapid clearance that restores plasma levels to baseline within 24 h [161,162]. In contrast, in vitro studies have demonstrated that effective concentrations of the predominant catechin, EGCG, range from 1 to 100 μmol/L [19].

A critical limitation to the expression of the otherwise numerous therapeutic effects of catechins is their insufficient in vitro stability. It has been observed that they are susceptible to degradation, oxidation, epimerization, and/or polymerization during the production, processing, and storage of phytoproducts or functional foods containing green tea [163,164]. Various factors significantly influence the chemical alterations of catechins. Their behavior in aqueous solutions is contingent upon their initial concentration, as they exhibit protective effects against their own oxidation. According to Krupkova et al. (2016), at micromolar concentrations, EGCG primarily degrades into dimeric forms, whereas at millimolar concentrations, a greater degree of epimerization to GCG occurs [165]. Additionally, it has been established that at temperatures below 44 °C and above 98 °C, degradation and epimerization, respectively, are the predominant factors compromising molecular stability [166]. The auto-oxidation of catechins in solution proceeds with the loss of hydrogen atoms, resulting in the formation of intermediate semi-quinone radicals, quinone oxidized products, and superoxide species. The degradation of catechins can be visually detected through a noticeable color change in the solution, transitioning from transparent to brown due to the polymerization of catechin dimers [165]. The stability of catechins also exhibits a decreasing trend at pH levels exceeding 4, with the highest instability observed at pH values greater than 8 [164,167]. Elevated oxygen levels in the environment, in the absence of antioxidants, further accelerate the oxidation rate of catechins [164]. In addition, when catechin galloyl groups interact with metal ions, complexes are formed, compromising both their antioxidant activity and the absorption of metal ions in the body [168]. The incorporation of additives or stabilizers during the production process may also influence the stability of GTCs, underscoring the challenges inherent in processing these bioactive compounds [169].

Several pharmacokinetic characteristics significantly contribute to the low bioavailability of catechins:

- low chemical stability within the conditions of the gastrointestinal tract;
- extensive metabolism throughout various regions of the digestive system;
- poor intestinal permeability.

Although the absorption of catechins primarily occurs in the duodenum, the alkaline pH of the small intestine, coupled with the presence of reactive oxygen species, creates favorable conditions for the auto-oxidative reactions of catechins [161,170]. Furthermore, their activity is suppressed following interactions with digestive enzymes such as α-amylase, lipase, pepsin, and trypsin. Dietary proteins (excluding dairy proteins) and fats may also react with GTCs, resulting in precipitate formation (referred to as cream formation) [169]. Thus, it is recommended that catechins be consumed on an empty stomach [162,171]. Separately, there is an extensive intestinal metabolism, including phase II enzymatic reactions (glucuronidation, sulfation, methylation), which are additionally observed in the subsequent hepatic biotransformation. Some of the metabolites formed in the small intestine, along with a minor fraction of unchanged catechins, reach the colon, where they may undergo further metabolic degradation, including interactions with the gut microbiome [172,173]. It has also been established that the transport mechanism for available unchanged catechins across the gastrointestinal epithelium predominantly occurs via passive diffusion (including paracellular and transcellular routes), without the involvement of specialized transmembrane mechanisms. Simultaneously, they experience active efflux involving multidrug resistance proteins (MRP) and PgP, through which the majority of absorbed molecules are pumped back into the extracellular or intestinal space, further reducing their bioavailability [19]. 

Over the years, various strategies have been explored to overcome the challenges posed by the low stability and bioavailability of GTCs. One avenue investigated was the administration of excessively high doses of polyphenolic substances derived from green tea. Chow et al. (2003) suggested that multiple-dose administration of amounts equivalent to the EGCG content found in 8-16 cups of green tea daily could significantly saturate presystemic metabolism of orally administered polyphenols [162]. However, this approach has not garnered widespread acceptance, as it has been demonstrated that these substances, while being natural antioxidants, can also exhibit pro-oxidant properties. Both EGCG and other less effective catechins induce dose-dependent oxidative degradation of DNA, as well as the formation of hydroxyl radicals and superoxide anions [174]. Furthermore, preliminary studies have associated high-dose intake of diets enriched with 0.5–1% green tea polyphenols with hepatotoxicity, increased risk of intestinal inflammation, and nephrotoxicity. Galati et al. (2006) report that treatment of freshly isolated mouse hepatocytes with 200 μM EGCG induces dose- and time-dependent cytotoxicity, which correlates with the production of reactive oxygen species (ROS). Furthermore, the inclusion of glutathione (GSH), catalase, or ascorbic acid was found to reduce ROS levels and EGCG-mediated cytotoxicity [175]. In a separate study, Sang et al. (2005) found that after treating CF-1 mice with 400 mg/kg intraperitoneal EGCG, EGCG-2′-cysteine and EGCG-2″-cysteine metabolites were detected in the urine samples [176]. The authors suggest that these metabolites arise from the reaction of EGCG quinone intermediates with the thiol group of cysteine, supporting the hypothesis that high doses of EGCG may exhibit pro-oxidant effects in vivo. Additionally, Lambert and Elias (2010) observed that treatment of mice with high oral doses of EGCG resulted in dose-dependent hepatotoxicity, manifested as increased hepatic lipid peroxidation, expression of hepatic metallothionein I/II, and elevated hepatic levels of γH2A.X [177]. These findings further highlight the potential pro-oxidant effects of EGCG at high doses. Furthermore, human case reports have linked the consumption of green tea supplements to hepatotoxicity [178]. These cases typically involve doses ranging from 700 to 2100 mg/day and are associated with elevated serum transaminase and bilirubin levels, abdominal pain, and, in some cases, jaundice. Liver biopsies have shown portal and periportal inflammation and necrosis. The increased bioavailability of green tea catechins, in addition to individual patient sensitivity, is considered a contributing factor to these adverse effects [177]. Additional adverse effects include antithyroid activity, suppression of aromatase activity, digestive disturbances, malabsorption syndromes, and more [179]. Therefore, the effects of excessive intake of green tea polyphenolic compounds seem difficult to predict, and thus it has no practical value.

The protection of GTCs stability by incorporating additional ingredients during production and storage is a widely implemented approach. Ascorbic acid (Vitamin C) and sugars are naturally occurring components that are often added externally during the processing of green tea-based products [180]. In fact, ascorbic acid is a water-soluble antioxidant that possesses a broad range of bioactivities—it is essential for the development and maintenance of connective tissues, participates in the regulation of the nervous system, immune system, bone formation, wound healing, and many other physiological processes. Like catechins, it exhibits anti-inflammatory and anticancer activities [181,182]. The protective properties of ascorbic acid concerning the predominant catechin, EGCG, are attributed to its reversible conversion to the oxidized product, dehydroascorbic acid. This conversion inhibits the oxidation of EGCG to EGCG-quinone and other intermediate products. Additionally, both ascorbic acid and dehydroascorbic acid inhibit the hydrolysis of catechins. According to Komatsu et al. (1993), the inhibition of isomerization increases with the concentration of the additive in the catechin mixture [182]. In this regard, Chen et al. (2020) noted that this statement is true until a critical concentration of 10 mmol/L (at 80 °C) is reached, as higher concentrations of ascorbic acid can actually promote catechin degradation, demonstrating its dual nature [163]. However, in vivo studies have shown that the absorption of EGC and EGCG is significantly enhanced when treated with formulations containing both ascorbic acid and sucrose. In addition to the aforementioned catechins, this combination also increases the accumulation of ECG in Caco-2 cells [161]. Even greater digestive stability of catechins has been achieved with formulations containing ascorbic acid and xylitol, which is particularly suitable for patients suffering from diabetes [183,184,185]. In their study, Son et al. (2016) found that the digestive stability of GTCs in this latter combination is further enhanced by encapsulation in γ-cyclodextrin or coating with hydroxypropyl methylcellulose phthalate [186]. Furthermore, it is suggested that other sugars (such as fructose, glucose, mannose, and galactose) also confer a protective effect on EGCG by reducing oxygen solubility, chelating transition metal ions, and scavenging reactive oxygen species in the environment [187,188]. Molecular modification of green tea catechins may also serve to enhance their bioavailability. The strategy of biological activation, frequently employing esterases, has a well-established history in the pharmaceutical development of prodrugs [189]. For instance, protecting the reactive hydroxyl groups of EGCG by converting it into an acetylated derivative results in not only greater stability under mildly alkaline physiological conditions compared to its free form but also may improve its biological activity. Lam et al. (2004) noted increased efficacy in proteasome inhibition and cell death induction, while Landis-Piwowar et al. (2007) presented evidence that treating cultured human MDA-MB-231 breast cancer cells with EGCG acetate led to higher intracellular accumulation of the active compound, accompanied by increased proteasome inhibition, reduced growth, and induced apoptosis [190,191]. According to Wang et al. (2013), this modified form also inhibits the growth, development, and angiogenesis of experimental endometriosis in mice [192]. An in vivo study revealed that acetylated EGCG exhibits a stronger ability to inhibit the proliferation and metastasis of breast cancer cells compared to EGCG [193]. On the other hand, the in vitro research conducted by Zhang et al. demonstrates unsatisfactory results regarding the antioxidant activity of two types of mono-substituted acetylated EGCG, three types of substituted acetylated EGCG, and eight types of substituted acetylated EGCG. Through their study, they reaffirm the observation that the antioxidant efficacy of phenolic compounds is primarily determined by the quantity and positioning of hydroxyl groups. Consequently, as the number of acetylated sites within the EGCG molecule increases, the overall antioxidant capacity, the superoxide anion radical-scavenging ability, and the hydroxyl radical-scavenging ability decrease [194]. The same conclusions were drawn in the investigation of EGCG–laurate [195]. Meanwhile, another of the few in vivo studies investigating the efficacy of chemically modified GTCs reports that the antioxidant capacity of acylated EGCG derivatives depends on the type and position of the substituents. Ester derivatives of EGCG with stearic acid, eicosapentaenoic acid, and docosahexaenoic acid not only exhibit enhanced lipophilicity but also demonstrate superior antioxidant activity in scavenging the 1,1-diphenyl-2-picrylhydrazyl radical compared to native EGCG [196]. Apparently, chemical modification and the reduction of free hydroxyl groups in the EGCG structure can also alter its antibacterial properties, as a gradual increase in antibacterial activity was observed with the elongation of the acyl chain in EGCG derivatives [197]. Among these derivatives, EGCG–palmitate demonstrated strong bactericidal effects against Gram-positive bacteria and rapidly eliminated methicillin-resistant *Staphylococcus aureus*. It has been found that certain ester derivatives can weaken the spore shell and disrupt its structural integrity, thus inhibiting spore germination [198]. In a similar manner, the introduction of fatty acid chains, which enhance the lipophilicity of EGCG and improve its permeability across cell membranes, likely alters its antiviral activity [199]. On the other hand, once the catechin is released from its precursor form, it undergoes identical biotransformation and efflux as free EGCG, explaining some authors’ desires to enhance the bioavailability of green tea catechins by suppressing their biotransformation. For example, simultaneous intragastric administration of 163.8 mol/kg EGCG and 70.2 mol/kg piperine in male CF-1 mice increased the plasma C_max_ and AUC of the catechin by 1.3 times. This observation is attributed to the inhibition of intestinal glucuronidation and gastrointestinal transit of the phenolic compound [200]. However, additional investigation is needed to fully elucidate the biological effects of these modifications in living systems. 

The pharmaceutical and food industries are constantly advancing, providing innovative approaches to enhance the stability and bioavailability of GTCs. Numerous studies demonstrate that nanostructure-based drug delivery systems provide protection against their physicochemical and biological degradation, facilitating their delivery to target sites within the organism. They can serve as delivery systems for both raw green tea extracts and purified catechins, while also contributing acceptable taste, appearance, and optimal rheological properties to the product [19,201]. Liposomes, which are surfactant-based nanovesicles (classified also as lipid-based carriers), represent a highly effective encapsulation system composed of phospholipids surrounding a core that contains bioactive compounds. They are biocompatible, biodegradable, non-toxic, and amphiphilic. Nano-liposomes offer improved stability of GTCs in alkaline solutions, leading to extended product shelf life and prolonged release, along with increased antioxidant functionality [202,203,204,205]. Ramadass et al. (2015) demonstrated that the combination of liposomal delivery of EGCG with paclitaxel further enhanced the bioavailability of the antitumor agent [206]. Phytosomes are similar structures in which plant extracts are dispersed within a phospholipid matrix. In this case, the active compounds are attached to the polar heads of the phospholipids in the micellar membrane [207]. Here again, the amphiphilic characteristics and emulsifying action of phospholipids enhance the absorption and bioavailability of the phytoconstituents. This approach results in higher biological activity while reducing dosage and prolonging the duration of action [207,208]. Niosomes represent a third type of surfactant-based nanovesicles, comprising a bilayer of nonionic surfactants and lipids (such as cholesterol). These systems demonstrate enhanced stability, reduced toxicity, and improved absorption of catechins compared to their free forms, while preserving their antioxidant capacity [209,210]. Shariare et al. (2020) developed a nanophytosome formulation of EGCG and egg phospholipid complex, which exhibits smaller particle size, higher drug-loading capacity, physical stability, and anti-inflammatory properties [207]. Green tea catechins can also be encapsulated within polysaccharide nanostructures, with chitosan being the most widely used material as a carrier. This is a natural, non-toxic, biocompatible, and biodegradable biopolymer known for its excellent film-forming properties [211]. It is utilized to produce carbohydrate-based carriers for local or oral administration. Various studies indicate that this approach enhances the bioavailability and plasma levels of catechins, such as (+)-catechin, (−)-epicatechin gallate, and EGCG, resulting in improved antioxidant, antibacterial, anti-cellulite, and other beneficial activities [212,213,214,215,216]. Coating chitosan nanoparticles with zein facilitates controlled release [217]. Additionally, the modification of chitosan particles loaded with EGCG using folic acid has been shown to enhance their inhibitory effect against MCF-7 human breast cancer cells [218]. Other polysaccharides that may also be employed in the formulation of functional foods and pharmaceuticals containing catechins include β-cyclodextrin, alginate, and gum Arabic [219,220,221,222,223,224]. In fact, some of these can be used in combination with chitosan. For example, Mohammadbaghban et al. (2024) demonstrated that the administration of catechin-loaded chitosan-alginate nanoparticles represents a beneficial therapeutic option against the behavioral and chemical alterations associated with Alzheimer’s disease in male Wistar rats [225]. 

Due to their functional and rheological characteristics, proteins represent a third type of agent suitable for nanodelivery of GTCs. Although interactions with them may partially reduce antioxidant activity, it is believed that they can protect catechins from degradation, thereby increasing their bioavailability [19]. Proteins, whether of plant or animal origin, are excellent candidates for encapsulating bioactive compounds due to their biodegradability, biocompatibility, and water solubility. Their hydrophobic regions facilitate interactions with poorly soluble catechins, while their hydrophilic domains enhance solubility in aqueous environments. This encapsulation protects active compounds from oxidation and enzymatic degradation in the gastrointestinal tract while also masking undesirable flavors. Furthermore, their ability to form gel-like structures upon ingestion enables controlled release, prolonging intestinal retention and enhancing absorption [226,227]. For instance, numerous studies have shown that proteins, especially those in milk, such as casein, are able to form complexes with catechins. As a result, the nanoencapsulation of EGCG within casein micelles preserves its bioaccessibility and anti-proliferative activity against colorectal cancer cells, maintaining efficacy comparable to its free form [228,229]. Whey protein fractions also show promise for encapsulating catechins. Nanoparticles made from heat-denatured β-lactoglobulin loaded with EGCG provide significant protection against oxidative degradation while suppressing the astringency and bitterness of the polyphenol [230]. Additionally, other protein-based nanocarriers, such as bovine serum albumin, have been used for sustained release of catechins, enhancing their therapeutic effects over time. For example, Yadav et al. (2014) used the desolvation method to load nanoparticles made from bovine serum albumin with catechin and epicatechin, resulting in improved stability at higher temperatures (37 to 57 °C) and slow, prolonged release, thereby enhancing the formulation’s efficacy against A549 cells [231]. Bovine serum albumin can also enhance the stability of EGCG. Li and Gu (2014) conjugated ovalbumin with dextran and further cross-linked it with glutaraldehyde, and the resulting conjugates were self-assembled with EGCG. This significantly increased the apparent permeability coefficient of the polyphenol across Caco-2 monolayers compared to an EGCG solution, suggesting that the demonstrated technique may improve its intestinal absorption [232]. Kumar et al. (2016) also explored the simultaneous use of proteins and polysaccharides in a single encapsulation system, demonstrating that a 3-day oral pre-treatment in mice with complexes of bovine serum albumin and chitosan significantly enhanced the radioprotective effects of the loaded green tea polyphenols by restoring redox status via the Nrf2-ERK pathway and reducing Bax expression [233]. Other proteins like soy protein, gelatin, bacterial cellulose-whey protein isolate, and corn zein are also potential candidates for producing protein-based carriers loaded with GTCs [234,235,236,237,238]. Emulsion-based nanodelivery systems have also been introduced as an option for enhancing the stability and bioavailability of catechins [169]. Peng et al. (2018) homogenized catechins with corn oil and polysorbate 80 to create an oil-in-water (O/W) nano-emulsion, achieving high storage stability over 20 days across various temperature conditions. In vitro simulated digestion assays showed increased bioavailability of EGCG compared to aqueous solutions. EGC, EC, and GCG exhibited significantly reduced bioavailability. On the other hand, the area under the plasma concentration-time curve (AUC_0–t_) for EGCG and EGC was increased in rats treated orally with the nano-emulsion [239]. Using similar techniques, catechins were loaded into a nano-emulsion made from soy protein and sunflower oil, resulting in nearly threefold increased bioavailability compared to the free form, with enhanced absorptivity in a Caco-2 cell model [240]. 

All these nanostructure-based drug delivery systems undoubtedly provide solutions for the continuous search for sustainable methods to incorporate GTCs into various functional foods (e.g., dairy products, baked goods, fruit beverages) or phytopharmaceuticals [169]. Despite their undeniable advantages, certain risks associated with the intake of these systems necessitate further and more in-depth studies. Primarily, the safety of nanomaterials used in formulations containing green tea or its extracts remains a topic of considerable debate. Preference is often given to biodegradable natural materials, although this does not diminish the need for toxicological vigilance [241]. A notable example is chitosan, which has been shown to cause cytotoxic effects at high systemic concentrations (some authors mitigate this through assembly with caseinophosphopeptides) [242]. Conversely, increased bioavailability of catechins raises the risk of reaching supratherapeutic plasma concentrations that may not be tolerated by the body, necessitating careful dosing to establish a safe threshold [169]. Additionally, more knowledge is required regarding potential interactions between GTCs and certain ingredients or medications. For example, cyclodextrins can be used as solubilizers and/or taste-masking agents in the production of high-catechin tea beverages, consumption of which could heighten the toxicity of P-glycoprotein substrates like digoxin [243]. Such studies focusing on interaction potential are crucial in the context of using GTCs as boosters or chemosensitizers.

## 7. Conclusions

Green tea exhibits a wide range of health benefits. The biologically active compounds present in green tea leaves are well-known and extensively studied. Some potential mechanisms of action of the tea catechins, which are considered the primary contributors to its biological activity, have been described. However, most data on the potential mechanisms of action of catechins are from in vitro studies, so the mechanisms of action of catechins in vivo are not yet fully understood. So, future research is needed to clarify the pharmacodynamics and therapeutic effects of catechins in humans. Furthermore, despite the potential health benefits of green tea, at present, catechins have limited application, primarily in tea beverages or dietary supplements. This limitation is attributed to issues such as low stability and poor oral bioavailability. Therefore, in order to fully exploit the beneficial effects of catechins, it is essential to develop effective methods to enhance their stability and bioavailability. On the other hand, catechins may also be involved in pharmacokinetic interactions with medications, which represents another significant concern regarding their use. Additionally, green tea catechins may be used as boosters or chemosensitizers which also poses risks for potential interactions. Currently, potential interactions between green tea/catechins and concomitantly administered medications are not well documented in humans and the available data are contradictory. Therefore, future studies about potential green tea/catechins interactions with certain medicines in humans are recommended in order to prevent serious adverse reactions.

In conclusion, despite the extensive information available on green tea and tea catechins to date, there remain numerous unknowns about the pharmacokinetics and pharmacodynamics of catechins. More knowledge is required regarding their efficacy and safety in vivo. Therefore, further research is needed to identify the optimal method for catechin intake and the realization of health-beneficial effects in humans.

## Data Availability

The original contributions presented in this study are included in the article. Further inquiries can be directed to the corresponding author.

## Abbreviations

The following abbreviations are used in this manuscript:

BAS	Biologically active substances
EGCG	(−)-epigallocatechin-3-gallate
ECG	(−)-epicatechin-3-gallate
EGC	(−)-epigallocatechin
EC	(−)-epicatechin
NF-kB	Nuclear factor kappa B
AP-1	Activator protein 1
MAPK	Mitogen-activated protein kinase
NRF2	Nuclear factor erythroid 2-related factor 2
STAT1	Signal transducer and activator of transcription 1
OATPs	Anion-transporting polypeptides
C_max_	Maximum plasma concentration
AUC	Area under the curve
P-gp	P-glycoprotein
CYP450	Cytochrome P450 enzymes
UGTs	Uridine 5′-diphospho-glucuronosyltransferases
CAT	Catalase
GSH-Px	Glutathione peroxidase
SOD	Superoxide dismutase
MDA	Malondialdehyde
ROS	Reactive oxygen species
RNS	Reactive nitrogen species
TNF alpha	Tumor necrosis factor alpha
ILs	Interleukins
ERK	extracellular signal regulated kinase
JNK	c-Jun N-terminal kinase
COX	Cyclo-oxygenase
LDL	Low-density lipoprotein
AMPK	Adenosine monophosphate-activated protein kinase
GTCs	Green tea catechins
TPS	Tea polysaccharides
